# Short-term effect of a developed warming moist chamber goggle for video display terminal-associated dry eye

**DOI:** 10.1186/s12886-018-0700-y

**Published:** 2018-02-07

**Authors:** Yueping Ren, Jie Chen, Qinxiang Zheng, Wei Chen

**Affiliations:** School of Ophthalmology and Optometry, Wenzhou Medical University, 270 Xueyuan West Road, Wenzhou, Zhejiang 325027 People’s Republic of China

**Keywords:** Dry eye, Video display terminal, Warming moist chamber goggles, Tear film

## Abstract

**Background:**

Video display terminal (VDT)-associated dry eye (DE) patients are the rising group worldwide, and moisture goggles are the preferable treatment since they are capable of improving tear film stability and DE discomfort. The current study aims to evaluate the short-term efficacy and safety of the developed warming moist chamber goggles (WMCGs) for VDT-associated DE patients.

**Methods:**

In this prospective self-control study, 22 DE patients (22 eyes) working with VDTs over 4 h daily were enrolled and instructed to wear WMCGs for 15 min. Sodium hyaluronate (SH, 0.1%) eyedrops were applied as a control on another day on these same patients, however 4 subjects denied the eyedrop application. The symptomatology visual analog scale (VAS) score, tear meniscus height (TMH), noninvasive tear film break-up time (NI-BUT), tear film lipid layer thickness (LLT), and bulbar conjunctival redness were assessed with Keratograph 5 M at baseline, 5, 30 and 60 min after treatment. The WMCGs wearing comfort was also evaluated.

**Results:**

The ocular discomfort evaluated by VAS decreased in the WMCGs group throughout 60 min (*P*<0.001), better than the control group levels (*P* ≤ 0.015). TMH, NI-BUT (including the first BUT and average BUT) increased than baseline level accross 60 min in the WMCG group (*P* ≤ 0.012), while those in the control group only showed temporary improvements in 5 min. LLT also increased obviously after WMCGs wear, while the change in the control group was nearly innoticeable. No adverse responses were detected.

**Conclusions:**

Temporary use of the WMCGs is able to relieve ocular discomfort, and improves tear film stability in DE patients for at least 1 h, making it a promising alternative to other treatments.

## Background

In modern society, some external and occupational environments can be risk factors for the development of dry eye (DE), including low relative humidity, high wind velocity, air conditioning, and long time working with video display terminals (VDTs) [1–4]. Excessive tear evaporation and tear film instablity can be caused by these conditions and induce dry eye symptoms, such as eye dryness, burning, gritty, itchy, scratchiness, soreness, and blurred vision [5]. Nearly 13.1% of the middle-aged Japanese VDT users experience dry eye symptoms in a large sample investigation [6]. Excessive VDT use results in decreased blink rates, which then delay the coating of the meibomian gland-sourced lipid layer over the aqueous layer, and consequently increase tear evaporation rates [7]. The instable supply of the lipid layer plays a vital role in the development of DE in VDT users. The VDT-associated dry eye patients is an increasing DE group in the modern world, suffering from eye discomfort or even mental fatigue and headache [8].

To increase the lipid layer thickness (LLT) is the suggested way to decrease tear evaporation and stablize the tear film in DE patients with lipid layer malfunction [9, 10]. Different methods have been used in clinic for the purpose of thickening lipid layer including manual massage, heat compress, various types of goggles and the rising therapy of intense pulsed light (IPL) [11, 12]. The traditional heat compress is usually inconvenient with poor patient compliance, while the IPL therapy is currently cost-prohibitive and still need more investigations. Moisture goggles or eyelid warming devices are reported to be capable of improving tear film stability, lipid layer thickness, as well as dry eye symptoms and ocular comfort [9, 13–15]. Besides, healthy subjects also benifit from these devices since 10 mins treatment of a eyelid-warming device could provided more effective warming and ocular surface comfort than warm compresses [16]. However, the previous studies usually focused on the effects of the devices on meibomian gland dysfunction (MGD) patients, and little results were available for VDT-associated DE. Thus in the current study, we develped a new warm moist chamber goggles (WMCGs) to deliver heat to the periocular area and increase the humidity by attaching moist sponges in a sealed periocular chamber, and evaluated the short term effects of this device on VDT-associated DE subjects, including DE discomfort relief, the changes of noninvasive tear breakup time (NI-BUT), tear meniscus height (TMH) and LLT measured by Keratograph 5 M, compared with the results of the 0.1% sodium hyaluronate (SH) eyedrops.

## Methods

### Subjects

In this prospective self-control study, a total of 22 DE patients (22 eyes) aged at 26.5 ± 4.85 years were enrolled in the Ocular Surface Center of the Eye Hospital of Wenzhou Medical University. The use of the chamber goggles is currently a part of standard care for DE patients, and the whole procedure of the study was approved by the Institutional Review Board of Wenzhou Medical University and adhered to the tenets of the Declaration of Helsinki.

Written informed consent was obtained from all subjects before participating in the study. The inclusion criteria consisted of DE symptoms persisting for at least 3 months with two or more than two kinds of symptoms such as irritation, eye desiccation, burning sensation, redness, reduced visual acuity, ocular pain, double vision, eyestrain, or tiredness; working in offices with VDTs more than 4 h everyday, since long time of VDT work (more than 4 h daily) was observed to be associated with a high incidence of DE disease [6, 17]; an OSDI (ocular surface disease index) over 13 [18, 19]; a fluorescein TBUT of < 5 s or a Schirmer I test score of ≤ 5 mm/5 min; no use of topical anti-inflammatory drugs (i.e. 0.05% cyclosporine A or steroids) except for artificial tears that has been stopped for at least 1 week. Patients with a history of ocular surgeries, any ocular diseases, contact lens use, any systemic diseases or medication, or perimenopausal, pregnant and lactating women were excluded. For every enrolled patient, both eyes received 0.1% SH eyedrops and/or WMCGs wear, however only the eye with smaller TBUT, or smaller Schirmer I test score if the TBUT is the same in both eyes, or the right eye if both TBUT and Schirmer I test score are the same in both eyes, was selected for statistical analysis.

### Study design

A same doctor conducted the enrollment tests of the DE patients on the first day. They were asked to return to the study center to apply 0.1% SH eyedrops (Santen, Osaka, Japan) in both eyes for one time at 9 AM on the second day, and wear a pair of WMCGs for 15 min from 9 AM on the third day. The ambient temperature and humidity in the examination room were monitored and maintained at a range from 23 to 24 °C and 30 to 40% respectively, and the patients need to stay in the room for 20 min before treatments. Clinical outcome measures and subjective effects were assessed in the both eyes at baseline, 5, 30 and 60 min after eyedrop instillation or WMCGs wearing, including the visual acuity, the intraocular pressure (IOP), TMH, NI-BUT, tear film lipid layer, and bulbar conjunctival redness with Keratograph 5 M (K5M; Oculus Optikgeräte GmbH, Wetzlar, Germany), as well as OSDI scores. The safety and wearing comfort were also evaluated for the treatment with the WMCGs. The comparisons of the subjective and objective therapeutic effects of the two treatments were made breadthwise and lengthwise. The patients were asked to get up before 7 AM to minimize the influence of longtime eye closure on tear film [20], and avoid eye makeup and swimming recently.

#### Subjective symptoms evaluation

DE symptoms were evaluated according to the OSDI questionnaire by DEWS [21, 22]. Visual Analog Scale (VAS) were used to assess the degree of subjective discomfort [23]. Subjects were required to grade ocular discomfort from the baseline on a 0 to 10 scale, where 0 was no discomfort and 10 was the most discomfortable. Higher scores on the visual analog scale referred to a greater feeling of discomfort in the eyes in this study. Subjects were also inquired about their wearing comfort after WMCGs wear.

#### Tear meniscus height

Objective tear function parameters were measured with the Keratograph 5 M in a darkened room. Lower tear film meniscus images were captured with the Keratograph 5 M equipped with a modified tear film scan software. TMH was measured with an integrated ruler on the vertical line through the pupil center.

#### Noninvasive tear film break-up time

The Keratograph 5 M automatic infrared illumination was deactivated, and the Placido rings were projected onto the cornea. After adjusting the focus, subjects were required to blink twice and to keep their eyes open for as long as possible during the NI-BUT measurements. Tear film topographic data were continuously recorded and discontinued at the next blink. In this study, first break-up time (FBUT) was defined as the time in seconds between the last complete blink before detection and the first distortion in the individual ring. The average break-up time (ABUT) is the mean time of the break-up timepoints during the whole detecting period.

#### Corneal fluorescein staining

Corneal fluorescein staining (CFS) was evaluated by instilling 0.5 ml of 5% fluorescein solution into the inferior conjunctival sac using a micropipette. The cornea was examined using slit-lamp microscopy in cobalt blue light 3 min after fluorescein instillation. The staining in five different regions was assessed according to a standardized grading system ranging from 0 to 4 [24]. The total scores were used as an index of epithelial integrity.

#### Lipid layer evaluation

The white interference light was projected onto the tear film. The colors and structures of lipid layer were observed and video recorded in real time between multiple blinks. During the video recording, subjects were instructed to blink naturally. Analysis of the lipid layer was achieved by evaluating color interference patterns in zones of specular reflection at the air/lipid layer. If colors and structures are visible, the lipid layer is thick. If there are neither colors nor structures visible, the lipid layer is very thin.

#### Bulbar Redness index (BRI)

Nasal and temporal conjunctival bulbar areas were analyzed independently by the Keratograph 5 M. Subjects were instructed to open their eyes as widely as possible during the examination. Redness values were graded automatically ranging from 0 to 4 by the system. The final score was the average of the captured three values.

**Fig. 1:**
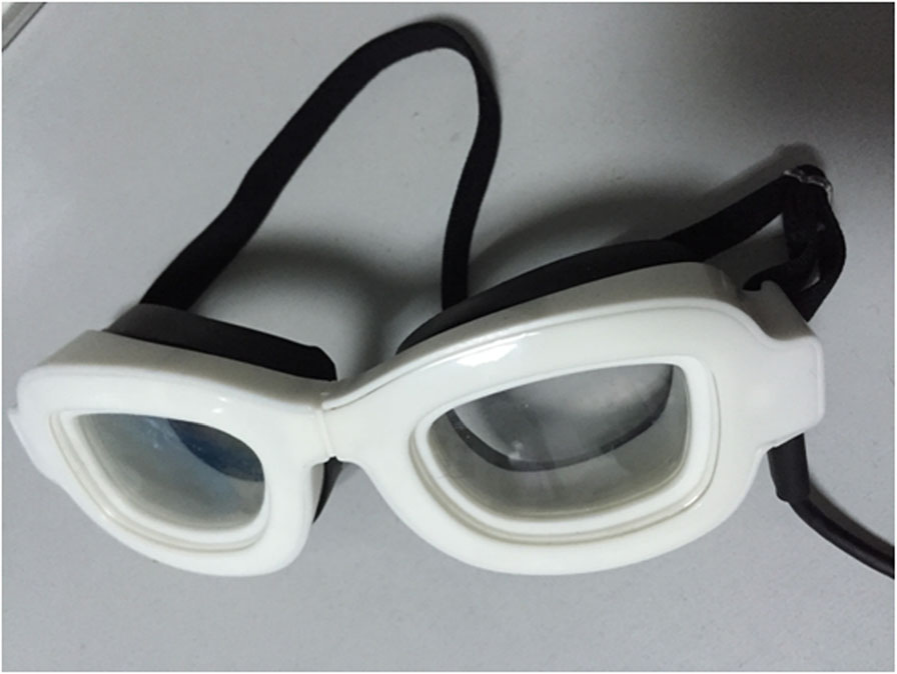
The photo of the new moist chamber goggle

The WMCGs used in our study is a pair of goggles designed to provide moisture and suitable heat to the periocular region (Fig. 1). The heat is produced by the installed heat elements. The WMCGs need preheating for 6 min, then moisture the attached sponges with 1 ml 0.9% sterile saline. Wear the goggle for 15 min when the light turned from red to blink. The local temperature maintained at 40 °C, and the treatment would stop when the light turned off. And the subjects were inquired about their satisfaction with the WMCGs wearing, which was reported as very comfortable, comfortable, or uncomfortable.

#### Statistical analysis

Statistical analysis was performed using the SPSS software version 20.0 (SPSS Inc., Chicago, IL). Comparisons of the subjective and objective parameters between the two methods of treatment at baseline, 5 min, 30 min and 60 min apply the independent t test if the data is of the normal distribution, or otherwise the Mann-Whitney U rank test. Comparisons of the data between each time point (15, 30, and 60 min) and baseline were conducted using the paired t test if the data is of the normal distribution, or otherwise the Wilcoxon signed rank test with the Bonferroni correction. Two-tailed *P* < 0.05 were considered to indicate a statistically significant difference.

## Results

In this prospective self-control clinical study, 22 VDT users with dry eye in both eyes (14 women and 8 men; mean age, 26.5 ± 4.85 years) were recruited to evaluate the effect of WMCGs on tear functions, compared with those of once use of 0.1% SH eyedrops. However 4 subjects (2 men and 2 women) denied to apply 0.1% SH eyedrops and only finished the tests of WMCGs, making the control group 4 cases less than the WMCGs group. Table 1 shows the comparison of clinical characteristics of the involved subjects at baseline between the WMCGs group and the control 0.1% SH group. No significant differences were found in age, sex distribution, the time of VDT use, OSDI score, or the tear function parameters between the two groups (*P* ≥ 0.244).

**Table 1:** The pretreatment clinical characteristics of the subjects in the WMCGs and the 0.1% SH groups

	WMCG group (*N* = 22)	0.1% SH group (*N* = 18)	
	Median (25%,75%)/	Median (25%,75%)/	*P*
	Mean ± SD	Mean ± SD	
Age (Year)	26.5 (25, 28.25)	26 (25, 27.25)	0.510
Gender (M:F)	8:14	6:12	0.968
VDT (h)	9.41 ± 1.87	9.79 ± 2.01	0.570
FBUT (s)	3.49 ± 0.97	3.52 ± 1.22	0.665
ABUT (s)	4.86 ± 1.34	4.92 ± 1.69	0.936
CFS	1 (0,3.25)	0 (0,2)	0.244
SIt (mm)	16.49 ± 10.98	15.63 ± 11.69	0.824
IOP (mmHg)	14.51 ± 2.38	15.00 ± 2.18	0.434
OSDI	37.49 ± 15.24	36.93 ± 16.69	0.918

*WMCGs* warming moist chamber goggles, *SH* sodium hyaluronate, *VDT* video display terminals, *FBUT* first break-up time, *ABUT* average break-up time, *CFS* corneal fluorescein staining, *SIt* Shirmer I test, *IOP* intraocular pressure, *OSDI* ocular surface disease index

### Subjective VAS score and wearing satisfaction

**Fig. 2:**
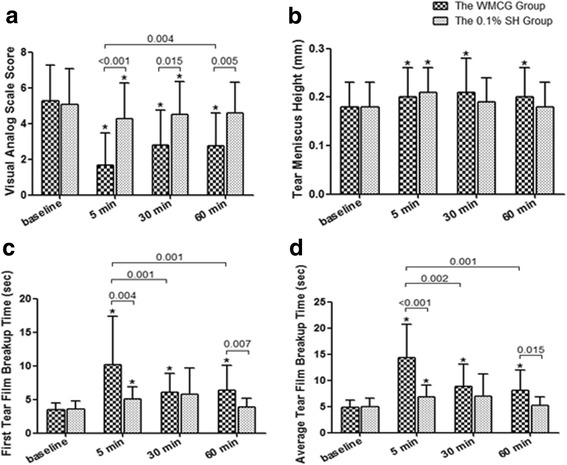
The changes of VAS, TMH, FBUT and ABUT after treatment in the WMCGs and the 0.1% SH groups. The VAS (**a**), TMH (**b**), FBUT (**c**) and ABUT (**d**) improved significantly across 60 min after wearing goggles for 15 mins (*P* ≤ 0.012); however the improvement only existed for these parameters at 5 min after 0.1% SH application in the control group (*P* ≤ 0.011), as well as for VAS at 30 min (*P* = 0.017). The difference bwtween the two groups was significant for VAS across 60 min (*P* ≤ 0.015), for FBUT and ABUT at 5 min and 60 min (*P* ≤ 0.015), but bot for TMH (*P* ≥ 0.341). * Self-comparison (post-treatment values compared with baseline level) by paired t test with Bonferroni correction (*P* < 0.05)

Relief in ocular discomfort, which was evaluated by VAS, was observed after 5 min in the both groups. VAS in the WMCGs group decreased from 5.25 ± 2.00 at baseline to 1.68 ± 1.79 at 5 min after goggles wear (*P* < 0.001), and it kept significantly reduced throughout 60 min (*P* < 0.001); while in the control group, the improvement existed at 5 min (from baseline 5.07 ± 1.93 to 4.26 ± 2.02, *P* = 0.006) and 30 min (4.51 ± 1.83, *P* = 0.017) after 0.1% SH application, but not at 60 min (*P* = 0.058). The VAS was much lower in the WMCGs group than that in the control group across all the 3 timepoints (*P* ≤ 0.015) (Table 2, Fig. 2a). Wearing comfort of the WMCGs was reported as 27.27% (6/22) very comfortable, 68.18% (15/22) comfortable, and 4.55% (1/22) uncomfortable.

**Table 2:** The changes of VAS, TMH, FBUT and ABUT after treatment in the WMCGs and the 0.1% SH groups

	WMCG group (*N* = 22)		0.1% SH group (*N* = 18)		
	Mean ± SD	P*	Mean ± SD	P*	P**
VAS					
Pre-treatment	5.25 ± 2.00	N/A	5.07 ± 1.98	N/A	0.800
5 min post-treatment	1.68 ± 1.79	<0.001	4.26 ± 2.02	0.006	<0.001
30 min post-treatment	2.80 ± 1.96	<0.001	4.51 ± 1.83	0.017	0.015
60 min post-treatment	2.76 ± 1.84^a^	<0.001	4.60 ± 1.69	0.058	0.005
TMH					
Pre-treatment	0.18 ± 0.05	N/A	0.18 ± 0.05	N/A	0.701
5 min post-treatment	0.20 ± 0.06	0.004	0.21 ± 0.05	0.001	0.642
30 min post-treatment	0.21 ± 0.07	0.001	0.19 ± 0.05	0.275	0.341
60 min post-treatment	0.20 ± 0.06	0.012	0.18 ± 0.05	0.927	0.524
FBUT					
Pre-treatment	3.49 ± 0.97	N/A	3.52 ± 1.22	N/A	0.936
5 min post-treatment	10.20 ± 7.21	<0.001	5.10 ± 1.76	0.011	0.004
30 min post-treatment	6.06 ± 2.85^a^	0.001	5.74 ± 3.93	0.077	0.783
60 min post-treatment	6.34 ± 3.69^a^	0.001	3.87 ± 1.26	0.463	0.007
ABUT					
Pre-treatment	4.86 ± 1.34	N/A	4.92 ± 1.69	N/A	0.890
5 min post-treatment	14.29 ± 6.38	<0.001	6.86 ± 2.28	0.004	<0.001
30 min post-treatment	8.83 ± 4.31^a^	0.002	6.98 ± 4.29	0.069	0.228
60 min post-treatment	8.05 ± 3.89^a^	0.001	5.22 ± 1.60	0.566	0.015

*P*<0.05 was regarded as significant difference

*VAS* visual analog scale, *TMH* tear meniscus height, *FBUT* first break-up time, *ABUT* average break-up time, *WMCGs* warming moist chamber goggles, *SH* sodium hyaluronate

*Self-comparison (post-treatment values compared with baseline level) by paired t test with Bonferroni correction

**Comparison between the two groups at the same timepoint by independent t test

^a^Comparison between the value at 30 or 60 min and at 5 min after treatment

### Objective tear film functions

TMH increased significantly throughout the 60 min in the WMCGs group, which raised from 0.18 ± 0.05 at baseline to 0.20 ± 0.06 at 5 min (*P* = 0.004), and kept elevated at 30 min (*P* = 0.001) and 60 min (*P* = 0.012). However in the control group, the improvement only existed at 5 min after 0.1% SH application (*P* = 0.001). And no significant changes were observed in TMH across all timepoints between the two groups (*P* ≥ 0.341) (Table 2, Fig. 2b).

FBUT in the WMCGs group increased distinctively from 3.49 ± 0.97 at baseline to 10.22 ± 7.21 after 5 min (*P* < 0.001), and the improvement kept significant throughout 60 min (*P* = 0.001). In the 0.1% SH group, the increase is significant at 5 min (*P* = 0.011) but not in later timepoints (*P* ≥ 0.077). The difference between the WMCGs and the control groups is significant at 5 min and 60 min (*P* ≤ 0.007), but not at 30 min (Table 2, Fig. 2c). Similarly, ABUT in the WMCGs group also increased from baseline 4.86 ± 1.34 to 14.29 ± 6.38 after 5 min with a great raise (*P* < 0.001), which remained significant across 60 min (*P* ≤ 0.002). In comparison, ABUT also increased in the control group 5 min after 0.1% SH installation (*P* = 0.004), but the effect did not keep for 30 min (*P* ≥ 0.069). And significant difference was observed between the two groups at 5 min as well as at 60 min (*P* ≤ 0.015) (Table 2, Fig. 2d).

**Fig. 3:**
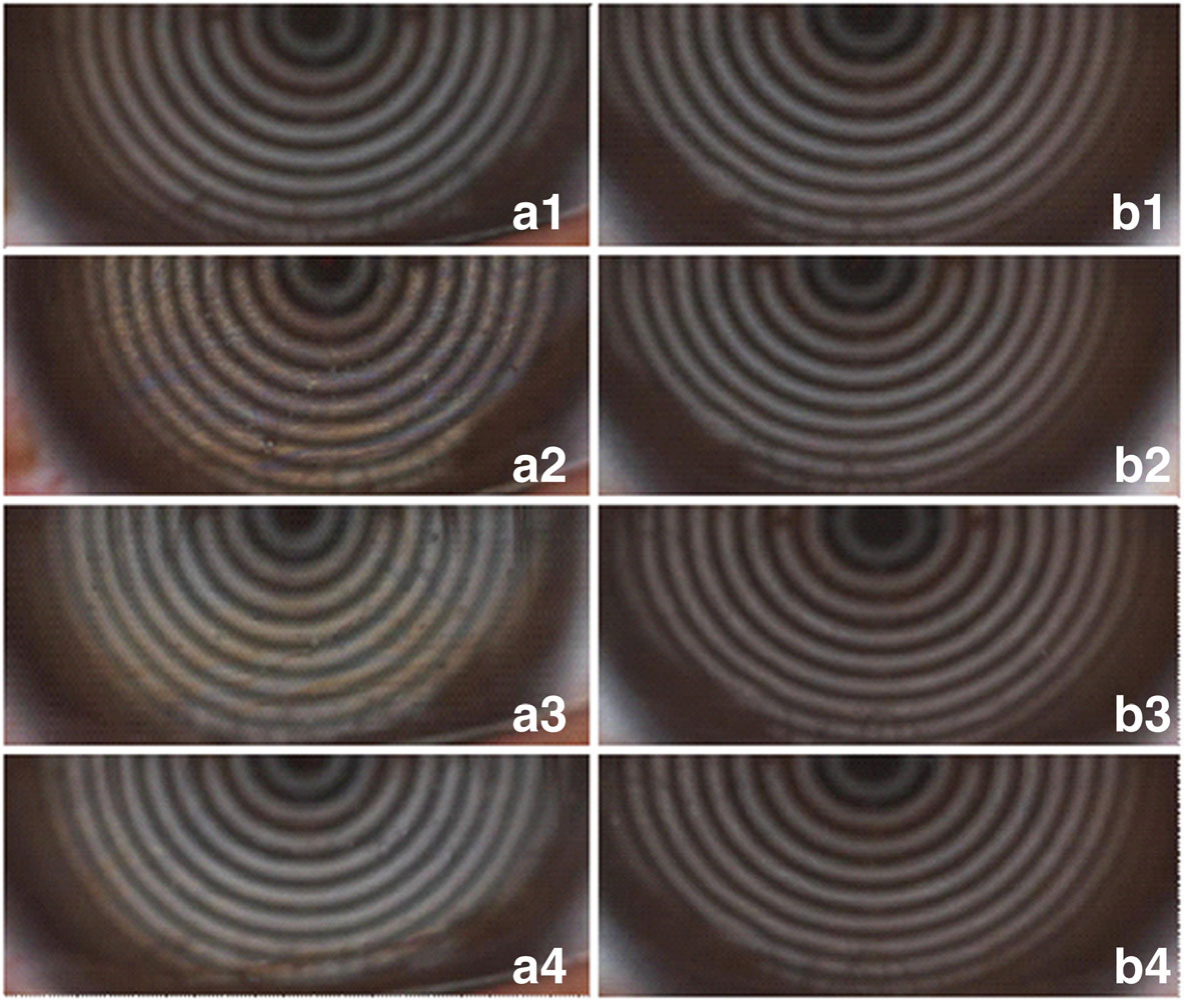
The representative pictures showing the improvements in lipid layer thickness after WMCGs wear. The lipid structure was very thin with pretty grey color and hardly visible at baseline (a1, b1). The LLT became much thicker with brown-blue interference patterns after 5 min of finishing WMCGs wear (a2), and the effect kept similar after 30 min (a3), and decreased but still thicker than the baseline level after 60 min (a4). However the effect in the control group was nearly unnoticeable accross 60 min (b2, b3, and b4)

Obvious improvements in LLT after wearing WMCGs were noted (Fig. 3). The lipid layer structure was very thin with pretty grey color, which was hardly visible at baseline. After 5 min of finishing WMCGs wear, the LLT became much thicker with brown-blue interference patterns in 13 (59.09%) eyes, which kept for 30 min and decreased gradually, however it was still thicker than the baseline level. In another 8 (36.36%) eyes, the lipid layer became thicker with brown color across 30 min, but it returned to the baseline level after 60 min. And no big difference in LLT was observed in the other 1 (4.55%) eye. However the effect in the control 0.1% SH group (18 eyes) was not comparable, since only 3 (16.67%) eyes has mild increase in LLT after 5 min, which turned unnoticeable after 30 min, and no changes was found in the other 15 (83.33%) eyes.

No ocular side effects were reported with the use of WMCGs or 0.1% SH eyedrops. There were no differences in the visual acurity (*P* = 0.957) and IOP (*P* = 0.531) before and after WMCGs wear. BRI also did not show significant difference at the 3 timepoints after treatment both in the WMCGs (*P* ≥ 0.151) and the control group (*P* ≥ 0.185).

## Discussion

In the current study, we used the developed warming moist chamber goggles to create a comfortable periocular condition in a sealed chamber, in which the temperature (40 °C) and humidity are well controlled. Constant and efficient eyelid warming melts the meibum oil and increases the secretion of meibum oil, which consequently improve the function tear film lipid layer. And the addition of moist sponges attached to the goggles produces steady water evaporation, which increases the periocular chamber humidity and provides a moist and warm ocular environment. Various types of eyelid warming or moisture devices have been shown to improve dry eye symptoms, ocular comfort, tear stability, and thickness of the tear film lipid layer in DE patients [9, 13–15]. And our WMCGs appear to have these positive effects as stated above.

Our results showed that ocular discomfort was significantly decreased in DE subjects after 15 min of WMCGs wear. The improvement in VAS lasted at least 60 min in the WMCGs group and the symptom relief was much better than the control group, indicating that the WMCGs is a preferable device for DE patients. In Korb er al.’s study, swim goggles improved DE ocular discomfort, however the symptoms returned in 91.9% of the patients 15 min after the goggles were removed [15]. Our WMCGs provide a longer period on DE symptom relief after goggle removal because their swim goggles don’t have the stable warming and moisture functions. However, the effect of mid/long term and mutiple use of the WMCGs needs to be investegated, to help to guide the standard use of the goggles.

The results of tear film parameters were in accord with the subjective symptom melioration. The advanced placido topograph Keratograph 5 M, is a recommended device to measure NI-BUT and TMH [25, 26], and it is also objective and reliable to score bulbar redness [27]. It has been a widely used tool to evaluate tear film and DE diagnosis in clinic [28]. In this study, we found that the TMH, NI-BUT and ABUT increased significantly 5 min after the application of WMCGs wear or the 0.1% SH eyedrops. However, the WMCGs wear has prolonged effect on tear film since these perameters were still better than the baseline level across 60 min while those in the control group failed to exert such effect. Bilkhu et al. reported that after a treatment period of an EyeBag eyelid warming device, significant improvements could be observed in TMH, BUT, LLT, ocular surface staining, and conjunctival hyperaemia in tested eyes compared with the contralateral control eye [29]. Simiar results has been demonstrated in other types of eyelid warming devices [9, 30], however these studies mainly focused on MGD patients while our results were obtained from VDT-associated DE patients, which is rarely discussed before.

Besides, the lipid layer thickness was significantly increased after WMCGs wear for 15 min in 95% subjects, and the improvement can last at least for 60 min. But the application of 0.1% SH eyedrops produced unnoticeable changes for lipid layer thickening. Tsubota & Nakamori demonstrated that the VDT use could induce a decreased frequency of blinking and an increased rate of tear evaporation, both of which contribute to DE development [31]. So the improvement of lipid layer thickness and stability is the core route to relieve or even get rid of DE symptoms. Tsubota’s team recently displayed that the use of a moist cool air device (MCAD) was able to improve BUT and tear evaporation rate, but not the lipid layer stability and corneal staining scores for VDT workers [32]. The core difference between their MCAD and our WMCGs is the control of the temperature, since they cooled the periocular area while we warmed this area to a steady temperature of 40 °C, which increases the lipid release and the lipid layer thickness, and subsequently decrease the tear evaporation. So our WMCGs is a better choice for VDT-associated DE patients.

The safety and wearing comfort have been approved by the subjects, and no adverse events were reported during this study. Our WMCGs deliver heat through a electrocircuit which is insulated in the goggle frame, and the maximal power of the electrocircuit can only reach 40 °C, so the safety of the WMCG is well guaranteed. This study aims to show the short-term effect of once use of the WMCGs in the VDT-associated DE patients, while the long-term results with repeated use of the goggles would be provided in our further studies, as well as in the other types of DE patients like the MGD or Sjögren syndrome patients.

## Conclusions

This study demonstrates that the short-term use of the developed WMCGs is able to improve ocular comfort, and increase TMH, NI-BUT and LLT in VDT-associated DE patients. It is a better alternative for DE patients who refuse to use multiple eyedrops or who have unfavorable effects of eyedrops. The environmentally friendly nature of the WMCGs would also be highly evaluated since it is resource-saving and can be used repeatedly for a long period.

## Abbreviations

ABUT: Average break-up time
BRI: Bulbar Redness index
DE: Dry eye
FBUT: First break-up time
IOP: Intraocular pressure
IPL: Intense pulsed light
LLT: Lipid layer thickness
MCAD: Moist cool air device
MGD: Meibomian gland dysfunction
NI-BUT: Noninvasive tear film break-up time
OSDI: Ocular surface disease index
TMH: Tear meniscus height
VAS: Visual analog scale
VDT: Video display terminal
WMCGs: Warming moist chamber goggles
